# Selective Oxygenation of 3,4‐Dihydro‐2*H*‐Pyran to 5,6‐Dihydro‐2*H*‐Pyran‐2‐One With Pd/C Catalyst and Molecular Oxygen

**DOI:** 10.1002/cssc.202502360

**Published:** 2026-03-29

**Authors:** Naoki Kawabata, Mizuho Yabushita, Keiichi Tomishige, Yoshinao Nakagawa

**Affiliations:** ^1^ Department of Applied Chemistry Graduate School of Engineering Tohoku University Sendai Miyagi Japan; ^2^ Research Center for Rare Metal and Green Innovation Tohoku University Sendai Miyagi Japan; ^3^ Advanced Institute for Materials Research (WPI‐AIMR) Tohoku University Sendai Miyagi Japan

**Keywords:** allylic oxidation, biomass, furfural, heterogeneous catalysis, palladium

## Abstract

The oxidation of 3,4‐dihydro‐2*H*‐pyran (DHP), which can be produced from lignocellulose‐derived furfural, was investigated with various catalysts and molecular oxygen as an oxidant in a H_2_O solvent. 5,6‐Dihydro‐2*H*‐pyran‐2‐one (DHPO) was obtained in moderate yield with a carbon black‐supported Pd catalyst (Pd/C). The addition of base (NaHCO_3_) suppressed the side reaction of DHP (i.e., hydration of DHP) and increased DHPO yield up to 52%. The activity of the catalyst was slightly decreased after three‐time use but recovered by the heat‐treatment under H_2_. Through the investigation of various Pd/support catalysts, the catalysts with low valent and stable Pd^0^ phase during the reaction, such as Pd/C and Pd/BN, showed high activity in DHPO formation. Positive and almost zero reaction orders were observed with respect to O_2_ pressure and DHP concentration, respectively, indicating the involvement of oxygen species in the rate‐determining step and the strong adsorption of DHP with catalyst. The addition of radical scavenger lowered the formation amount of DHPO, suggesting that a radical intermediate is involved in the reaction.

## Graphical abstract

Selective oxygenation: The oxidation of 3,4‐dihydro‐2*H*‐pyran, which can be produced from lignocellulose‐derived furfural, was investigated with various catalysts using O_2_ as an oxidant in a H_2_O solvent. 5,6‐dihydro‐2*H*‐pyran‐2‐one, a value‐added product, was obtained in 52% yield with reusable Pd/C catalyst.

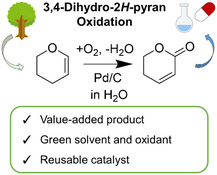

## 1 Introduction

**SCHEME 1:**
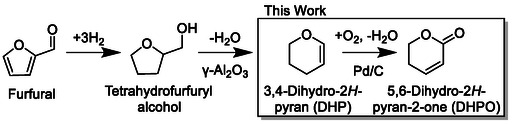
Synthesis of DHP from furfural and this study.

Synthesis of chemical products from biomass resources has attracted attention due to concerns about the depletion of petroleum resources. In particular, lignocellulosic biomass consisting of cellulose, hemicellulose, and lignin is a promising alternative resource due to its abundance and renewability. Because of the complex nature of biomass, the conversions of biomass to chemicals usually start with the production of small molecules called platform chemicals by thermal, chemical or biological processing [1, 2, 3]. Among these platform chemicals, furfural is an especially important one, which is produced from acid‐catalyzed dehydration of hemicellulose [4, 5, 6]. Furfural is industrially produced in excess of 300,000 tons per year, and its derivatives are widely used as industrial solvents, disinfectants, fuel additives, pharmaceuticals, plastics and so on [5, 6, 7]. Among these derivatives, 3,4‐dihydro‐2*H*‐pyran (DHP) is produced in high yield (∼90%) by dehydration of tetrahydrofurfuryl alcohol [8, 9, 10, 11], which can be quantitatively obtained from furfural by hydrogenation [12, 13] (Scheme 1), and therefore, DHP is a potential platform chemical. For example, it has been reported that 1,5‐pentanediol can be produced from DHP by hydration to 2‐hydroxytetrahydropyran followed by hydrogenation [9]. Meanwhile, oxidative conversion is also an attractive method when a green and inexpensive oxidant is employed. Therefore, we focus on oxidation of DHP to obtain useful chemical products.

The previous studies on the oxidation of DHP are summarized in Supporting Information Table S1. The main oxidation products are δ‐valerolactone [14, 15, 16, 17], glutaric acid (GA) [18], and 5,6‐dihydro‐2*H*‐pyran‐2‐one (DHPO) [19, 20, 21, 22]. Among these products, δ‐valerolactone and GA can be produced even from other biomass‐derived platform chemicals, lowering the necessity of their production from DHP. For example, it has been reported that δ‐valerolactone can be produced by dehydrogenation of 1,5‐pentanediol [23], hydrogenolysis of furoic acid [24, 25], and Baeyer–Villiger oxidation of cyclopentanone [26]. All these substrates can be synthesized from furfural. GA can be also produced from cyclopentanone by oxidation with H_2_O_2_ [27, 28]. On the other hand, it is difficult to produce DHPO from biomass‐derived compounds other than DHP. In addition, DHPO is a fine chemical that is used as material for functional polymer [29] and expected to be an intermediate for pharmaceuticals [30]. Chidambaram et al. reported the oxidation of DHP with pyridium dichromate (PDC)*/t*‐BuOOH to produce 56% yield of DHPO, but this method used expensive and toxic oxidant (PDC) and nongreen solvent (dichloromethane) [21]. Recently, Adbuh et al. have reported the oxidation of DHP with O_2_ as an oxidant in solvent‐free condition using a ZnFe_2_O_4_@γ‐Al_2_O_3_ catalyst [19]. The reported DHPO selectivity was 72% at 47% conversion (yield:35%). However, this report focused on the oxidation of tetrahydrofuran rather than DHP, and the details of catalysis were not investigated. Here, we investigated the oxidation of DHP with various catalysts using O_2_ as an oxidant in a H_2_O solvent, which is ranked as the greenest solvent [31, 32]. We found that DHPO is produced by the oxidation of DHP with a reusable heterogeneous Pd/C catalyst in high yield (52%).

## 2 Experimental

### 2.1 Catalyst Preparation

All the reagents used in the following procedure are listed in Supporting Information Table S2. The Pd/Support (Pd = 5 wt%) catalysts were prepared with the impregnation method as follows. Details of the supports are listed in Supporting Information Table S3. First, an aqueous solution of Pd(NO_3_)_2_ was added to a powdery support in a beaker until the surface of the support was saturated, and the mixture was stirred well with a glass rod. The beaker containing the wet sample was then placed on a hot plate, and the mixture was dried at 343 K. This process was repeated until the entire precursor solution was consumed. The solids were further dried in a drying oven at 383 K overnight. The dried catalysts except those using carbon‐based supports were calcined in air at 773 K for 3 h at a heating rate of 10 K min^−1^. They were reduced under a H_2_ flow at 473 K and passivated under a 2% O_2_/Ar flow at room temperature for 15 min. Pd/C catalysts were heated in a N_2_ flow instead of air at 773 K for 3 h at a ramp rate of 10 K min^−1^ and passivated under a 2% O_2_/Ar flow at room temperature for 15 min.

### 2.2 Activity Tests

The activity tests were carried out in a stainless‐steel autoclave (190 mL) equipped with a glass inner vessel. Substrate, water, and the passivated catalyst were put into the autoclave together with a spinner. After sealing, the reactor was filled with 0.8 MPa O_2_ (at r.t.), and the temperature, which was monitored with a thermocouple, started to rise. The reaction time of 0 h was defined as the time when the temperature reached 2 K lower than the target temperature, and the target temperature was maintained throughout the reaction. After an appropriate reaction time, the reactor was cooled in a cold‐water bath. The gas phase in the autoclave was collected into a plastic gas bag. The liquid phase was diluted with tetrahydrofuran and transferred to a vial. The autoclave itself was washed with tetrahydrofuran, and the liquid was combined to the reaction solution in the vial, and the catalyst was separated by filtration through a membrane filter if necessary.

Analysis of liquid phase was conducted with an HPLC (Shimadzu Prominence; Aminex HPX‐87H column (Bio‐rad), 0.01 M sulfuric acid as an eluent, RID) and a GC (Shimadzu GC‐2014) equipped with HP‐FFAP capillary column (diameter 0.25 mm, 30 m) and FID. A typical GC chart is shown in Supporting Information Figure S1. GC‐MS (Shimadzu GC‐MS‐QP2020NX, electron ionization (EI)) was also used for the identification of products (Supporting Information Figure S2). The gas phase was analyzed by using a GC with FID combined with a methanizer (Shimadzu GC‐2014, Porapak N packed column) and a GC (Shimadzu GC‐2014) equipped with HP‐FFAP capillary column. The conversion, selectivity, and carbon balance were calculated with the following equations

$$\mathrm{Conversion}\;\left(\%\right)=\frac{\text{Mole of substrate consumed}}{\text{Mole of input substrate}}\times 100$$

$$\mathrm{Yield}\;\left(\%\right)=\frac{\text{C-based mole of product}}{\text{C-based mole of input substrate}}\times 100$$

$$\text{Carbon balance}\;\left(\%\right)=\frac{\text{Sum of C-based}\text{ mole of products}+\text{C-based mole of residual substrate}}{\text{C-based mole of input substrate}}\times 100$$

Hot‐filtration test was carried out in the following procedure. The reaction was carried out in the same manner as above under the standard reaction condition for 16 h (reaction (i)). After the reaction, the liquid phase was collected with a syringe and filtered without cooling. Approximately 5 g of the filtrate was transferred to another reactor, and the reaction was carried out under pressurized O_2_ again for 8 h without catalyst (reaction (ii)). The liquid phases for both reactions (i) and (ii) were analyzed with the HPLC. Because the washing step of the autoclave in the collection procedure in the standard runs was skipped, there could be a small loss of material in the analysis.

Reuse test of the catalyst was carried out as follows. The reaction was carried out in the same manner as above for 20 h. Water was used as a diluting solvent for reaction mixture because the contact with tetrahydrofuran might change the surface state of Pd particles. The catalyst was separated from the reaction mixture by filtration, washed with water three times, dried on a hot plate set at 353 K overnight, and then used for the next run. After the 4th run, the collected and dried catalyst was treated with flowing H_2_ at 473 K for 1 h as regeneration.

### 2.3 Catalyst Characterization

A Rigaku MiniFlex600 diffractometer (Cu *K*α radiation) was used to measure the X‐ray diffraction (XRD) pattern of samples. Measurement conditions were step 0.02° and 10° min^−^^1^. For catalysts other than Pd/C(BP2000), crystallite size of Pd was calculated by Scherrer equation using the peak at 40°, while for Pd/C(BP2000), the peak at 47° was used. Scanning transmission electron microscope (STEM) images were taken with HITACHI HD‐2700. The catalyst was suspended in ethanol and was dropped on a Cu microgrid.

## 3 Results and Discussion

### 3.1 Oxidation of DHP With Various Catalysts

First, various catalysts which are generally used in oxidation reactions were tested for oxidation of DHP (Table 1). In the reaction without catalyst, DHP was easily hydrated, and 2‐hydroxytetrahydropyran (2‐HY‐THP) became the main product (Entry 1). In the presence of catalyst, other products were also formed such as DHPO (the target product), δ‐valerolactone (DVL) and its open‐chain form (5‐hydroxyvaleric acid; 5‐HVA), GA, and formic acid (FA). The yields of DVL and 5‐HVA are merged because they are interconvertible in the aqueous solution. DHPO was obtained in high yield only with Pd/C among the catalysts tested; however, the yield of DVL+5‐HVA was higher than that of DHPO. Pt/C, another noble metal catalyst, showed relatively high yield of GA (31%), which is the consecutive oxidation product of DVL and 5‐HVA (Entry 3). With non‐noble metal catalysts, the yields of DHPO, DVL+5‐HVA and GA were negligible, and large amount of 2‐HY‐THP remained. When H_5_PV_2_Mo_12_O_40,_ V_2_O_5_ and MnO_2_ were used, FA was produced as a characteristic product (Entries 5–7); however, the production of FA from more accessible substrates such as sugars has been already reported [33, 34]. From these results, Pd/C is a promising catalyst for the production of DHPO and thus was employed in the following investigation.

**TABLE 1:** The oxidation of DHP with various catalysts.

Entry	Catalyst	Conv. (%)	Yield (%)								Carbon balance (%)
			DHPO	2‐HY‐THP	DVL + 5‐HVA	GA	FA	CO	CO_2_	Others	
1	No catalyst	84	<1	75	<1	<1	<1	<1	<1	2	93
2	Pd/C(BP2000)	100	16	2	38	4	<1	<1	10	16	86
3	Pt/C	100	1	1	12	31	<1	<1	4	48	97
4	H_3_PMo_12_O_40_	84	<1	59	<1	<1	<1	<1	<1	10	87
5	H_5_PV_2_Mo_10_O_40_	88	<1	33	9	<1	7	<1	3	28	91
6	V_2_O_5_	95	<1	54	6	<1	4	<1	<1	30	100
7	MnO_2_	93	<1	51	4	<1	8	<1	1	26	97
8	CuO	95	<1	74	1	<1	1	<1	<1	4	85
9	Fe_3_O_4_	89	<1	76	<1	<1	<1	<1	<1	3	90

*Note:* Reaction conditions: heteropoly acid 110 µmol or metal oxide 1100 µmol as metal or Pt/C (Pt = 10 wt%) 0.050 g (26 µmol as Pt) or Pd/C(BP2000) (Pd = 5 wt%) 0.10 g (47 µmol as Pd), DHP 4.0 mmol, water 10 g, O_2_ 0.8 MPa, 353 K, 24 h.

Abbreviations: 2‐HY‐THP, 2‐hydroxytetrahydropyran; 5‐HVA, 5‐hydroxyvaleric acid; DHPO, 5,6‐dihydro‐2*H*‐pyran‐2‐one; DVL, δ‐valerolactone; FA, formic acid. Others, the products detected by HPLC other than listed ones; GA, glutaric acid.

### 3.2 Oxidation of 2‐HY‐THP With Pd/C

**FIGURE 1:**
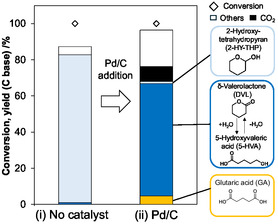
Oxidation of DHP with Pd/C catalyst after noncatalyzed hydration to 2‐hydroxytetrahydropyran (2‐HY‐THP). Reaction conditions: (i) DHP 4.0 mmol, water 15 g, O_2_ 0.8 MPa, 353 K, 24 h. (ii) Pd/C(BP2000) (Pd = 5 wt%) 0.10 g (47 µmol as Pd), liquid phase of run (i) 10 g, O_2_ 0.8 MPa, 333 K, 24 h.

Since the hydration of DHP to 2‐HY‐THP proceeded even without a catalyst, whether the hydration is desirable (within the reaction route toward DHPO) or not is important information for the improvement of reaction system of DHPO production. To examine the reaction pathway of DHP oxidation with the Pd/C catalyst, oxidation of 2‐HY‐THP with Pd/C catalyst was conducted (Figure 1). The reaction was carried out in two steps: (i) hydration of DHP without catalyst and (ii) oxidation of the product solution of (i) in the presence of the Pd/C catalyst. As a result, DHPO was not produced at all from 2‐HY‐THP, and instead, DVL+5‐HVA and GA were found to be major products. Therefore, 2‐HY‐THP is not a reaction intermediate of DHPO, and suppression of hydration of DHP is crucial to improve DHPO yield.

### 3.3 Suppression of Hydration of DHP by Addition of Base

**FIGURE 2:**
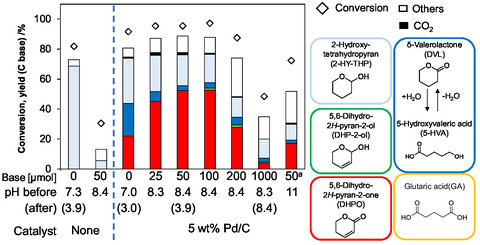
Effect of addition of NaHCO_3_ on the oxidation of DHP with Pd/C catalyst. Reaction conditions: Pd/C(BP2000) (Pd = 5 wt%) 0.10 g (47 µmol as Pd), NaHCO_3_ 0–1000 µmol or ^a^Na_2_CO_3_ 50 µmol, DHP 4.0 mmol, water 10 g, O_2_ 0.8 MPa, 313 K, 24 h. Detailed data are shown in Supporting Information Table S4.

In general, H^+^ catalyzes hydration of double bonds [35]. We thus added NaHCO_3_ as a base in an attempt to suppress the hydration of DHP in the Pd/C‐catalyzed oxidation of DHP (Figure 2; detailed data: Supporting Information Table S4). In the reaction without Pd/C catalyst, the suppression of hydration of DHP by the addition of NaHCO_3_ was observed. In the reaction with Pd/C, hydration was also suppressed and DHPO yield increased with the increase of NaHCO_3_ amount up to 50 µmol. However, when the amount of NaHCO_3_ was 200 µmol or more, DHP conversion and DHPO yield significantly decreased. When Na_2_CO_3_ was used as a stronger base than NaHCO_3_, DHPO yield also decreased. Therefore, we decided 50 µmol of NaHCO_3_ as the optimum amount and base. The pH values of the reaction solution were measured before and after the reactions. When 50 µmol of NaHCO_3_ was added, the decrease in pH was observed during the reaction because of the neutralization by the acid generated from DHP such as 5‐HVA. Meanwhile, in the case of 1000 µmol, the pH was maintained above eight, and the yield of DHPO was low. The results showed that DHPO formation was suppressed by basic conditions.

### 3.4 Support Screening of Pd Catalyst

The effect of support was investigated for Pd catalysts in the DHP oxidation in the presence of NaHCO_3_ (Table 2). All the Pd catalysts produced DHPO. Considering also the results in Table 1, Pd is the essential component in the DHPO production from DHP. The highest yield of DHPO was obtained with Pd/C(BP2000), while some other catalysts such as Pd/C(XC72R), Pd/BN, Pd/TiO_2_ and Pd/SiO_2_ gave comparable yields. We used Pd/C(BP2000) as the standard catalyst in this study, and this catalyst is simply denoted as “Pd/C”.

**TABLE 2:** DHP oxidation with various Pd/support catalysts.

Entry	Catalyst	Conv. (%)	Yield (%)								C. B. (%)
			DHPO	DHP‐2‐ol	2‐HY‐THP	DVL + 5‐HVA	GA	CO	CO_2_	Others	
1	Pd/C(BP2000)^a^	96	52	<1	21	3	<1	<1	1	11	93
2	Pd/C(Wako)^b^	84	22	1	13	7	<1	<1	1	33	94
3	Pd/C(XC72R)	98	50	<1	14	7	<1	<1	1	14	88
4	Pd/BN	98	48	<1	21	6	<1	<1	1	9	87
5	Pd/TiO_2_(P25)	96	35	4	19	2	<1	<1	<1	29	94
6	Pd/Al_2_O_3_	87	20	5	21	3	<1	<1	<1	22	85
7	Pd/ZrO_2_	97	38	<1	17	5	<1	<1	2	17	82
8	Pd/SiO_2_	92	31	2	29	5	<1	<1	1	10	85
9	Pd/CeO_2_	98	22	<1	35	4	<1	<1	<1	24	88

*Note:* Reaction conditions: Pd/Support (Pd = 5 wt%) 0.10 g (47 µmol as Pd), NaHCO_3_ 50 µmol, DHP 4.0 mmol, water 10 g, O_2_ 0.8 MPa, 313 K, 24 h.

Abbreviations: 2‐HY‐THP, 2‐hydroxytetrahydropyran; 5‐HVA, 5‐hydroxyvaleric acid; DHPO, 5,6‐dihydro‐2*H*‐pyran‐2‐one; DVL, δ‐valerolactone; FA, formic acid. Others, the products detected by HPLC other than listed ones; GA, glutaric acid.

a Standard Pd/C catalyst used in this study.

b Commercial Pd/C catalyst purchased from FUJIFILM Wako Pure Chemical.

### 3.5 Catalyst Performance

**FIGURE 3:**
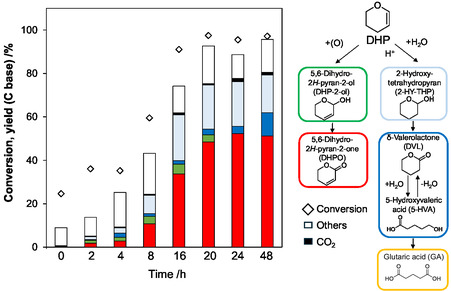
Time course of DHP oxidation with Pd/C catalyst. Reaction conditions: Pd/C(BP2000) (Pd = 5 wt%) 0.10 g (47 µmol as Pd), NaHCO_3_ 50 µmol, DHP 4.0 mmol, water 10 g, O_2_ 0.8 MPa, 313 K. Detailed data are shown in Supporting Information Table S6.

The time course of DHP oxidation with the Pd/C catalyst was investigated (Figure 3; detailed data: Supporting Information Table S5). The highest DHPO yield of 52% was obtained at 24 h. This yield value is higher than the highest reported one, 35% over ZnFe_2_O_4_@γ‐Al_2_O_3_ catalyst [19]. We prepared ZnFe_2_O_4_@γ‐Al_2_O_3_ catalyst with the reported procedure and carried out the activity test (Supporting Information Table S6 and Figure S3). We failed to reproduce the result: only 3% DHPO yield (59% conversion) was obtained in the conditions where 35% yield (at 49% conversion) was reported, and the blank run without catalyst in the same conditions gave similar results (2% yield at 58% conversion). In Figure 3, 5,6‐dihydro‐2*H*‐pyran‐2‐ol (DHP‐2‐ol), which is likely to be an intermediate of DHPO formation, was also produced at short reaction time. The decrease of DHPO from 24 to 48 h of reaction time was marginal, which means that the over‐oxidation of DHPO was very slow.

**FIGURE 4:**
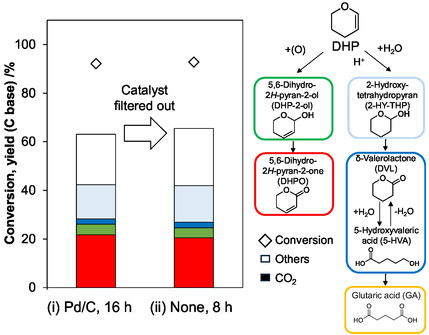
Hot‐filtration test of Pd/C catalyst. Reaction conditions: (i) Pd/C(BP2000) (Pd = 5 wt%) 0.10 g (47 µmol as Pd), NaHCO_3_ 50 µmol, DHP 4.0 mmol, water 10 g, O_2_ 0.8 MPa, 313 K, 16 h, (ii) liquid phase of run (i), O_2_ 0.8 MPa, 313 K, 8 h. *Note:* The product yields were lower than the standard runs because of the incomplete collection of the reaction mixture.

**FIGURE 5:**
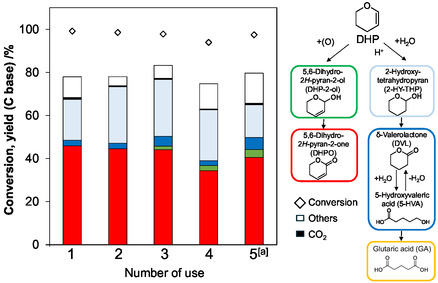
Reuse test of Pd/C catalyst. Reaction conditions: Pd/C(BP2000) (Pd = 5 wt%) 0.10 g (47 µmol as Pd), NaHCO_3_ 50 µmol, DHP 4.0 mmol, water 10 g, O_2_ 0.8 MPa, 313 K, 20 h. ^a^After 4th reaction, the catalyst was treated with flowing H_2_ at 473 K for 1 h. *Note*: The product yields were lower than the standard runs because of the incomplete collection of the reaction mixture.

The hot‐filtration test demonstrated that DHPO formation was almost stopped by the removal of Pd/C catalyst, suggesting the heterogeneous catalysis of Pd/C (Figure 4). The reusability of Pd/C catalyst was tested (Figure 5). The activity of the Pd/C catalyst was only slightly decreased after three‐time use and was recovered after the treatment of the spent catalyst with flowing H_2_ at 473 K. The total turnover number (TON = total amount (mol) of DHPO/input amount (mol) of Pd atoms) reached 1.8 × 10^2^ in the four runs. These tests demonstrated the stability of the Pd/C catalyst in the oxidation of DHP.

### 3.6 Characterization of Pd Catalysts

**FIGURE 6:**
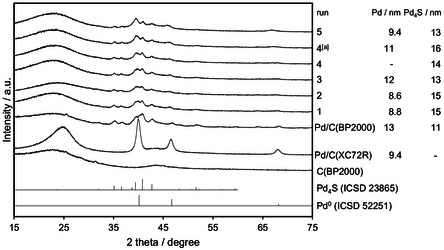
XRD patterns of Pd/C catalysts. The catalysts after reaction were those used in Figure 5. ^a^The catalyst after 4th reaction was treated with flowing H_2_ at 473 K for 1 h.

**FIGURE 7:**
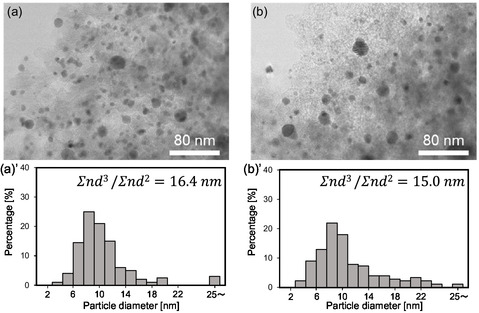
STEM images and particle size distribution of Pd/C catalyst (a) before reaction and (b) after 5th run of reaction (Figure 5).

Pd/C(BP2000) catalyst was characterized by XRD and STEM analyses (Figures 6 and 7). The XRD pattern of Pd/C catalyst displayed Pd_4_S and Pd^0^ phases even without prereduction. The peaks for Pd_4_S became weaker, while those for Pd^0^ became stronger upon the reuses of Pd/C(BP2000). These results indicated that the valence of Pd species remained low (∼0) in the reaction condition with pressurized O_2_, and the Pd_4_S phase was gradually changed to Pd^0^ phase. This sulfide phase is derived from the sulfur impurity in the carbon support (BP2000) [36, 37]. Another carbon‐supported catalyst, Pd/C(XC72R), did not contain Pd_4_S phase because of a lack of sulfur in XC72R but contained Pd^0^ as the only Pd‐related phase. The crystallite sizes of Pd and Pd_4_S phases were around 10 nm for both catalysts. Considering the similar performance of fresh Pd/C(BP2000), reused and regenerated Pd/C(BP2000) and Pd/C(XC72R), both Pd_4_S and Pd^0^ phases have similar catalytic activity. In the STEM image, metal nanoparticles with ≈5–15 nm in diameter were observed for both the fresh and spent Pd/C catalysts, agreeing with the XRD patterns.

**FIGURE 8:**
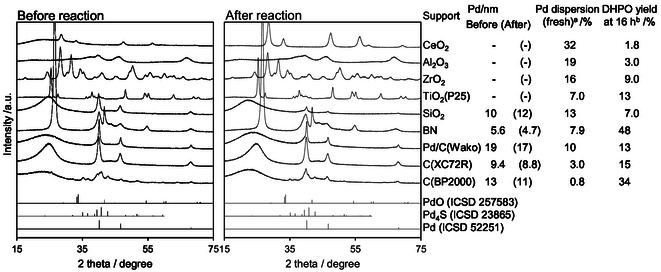
XRD patterns of Pd/Support catalysts before and after reaction. ^a^Determined by CO absorption. ^b^Reaction conditions: Pd/Support (Pd = 5 wt%) 0.10 g (47 µmol as Pd), NaHCO_3_ 50 µmol, DHP 4.0 mmol, water 10 g, O_2_ 0.8 MPa, 313 K, 16 h. Detailed data are listed in Supporting Information Table S7.

The relationship between the structure of Pd species and the catalytic activity for DHPO formation over supported Pd catalysts was then investigated (Figure 8, detailed data: Supporting Information Table S7). Because the formation amount of DHPO was not linearly increased with the reaction time (Figure 3), the accurate determination of reaction rate is difficult. We instead used the DHPO yield at 16 h of reaction time as an indicator of catalytic activity. We measured the CO adsorption amount to determine the Pd dispersion of fresh catalysts, and XRD patterns of fresh and used catalysts were acquired to estimate the crystallite size and oxidation degree of Pd species. The catalysts with a stable Pd^0^ phase during the reaction, such as Pd/BN and Pd/C(XC72R), gave high DHPO yields per Pd dispersion. Meanwhile, the catalysts which did not show Pd^0^ phase, such as Pd/CeO_2_, Pd/Al_2_O_3_, Pd/ZrO_2_, and Pd/TiO_2_, gave low DHPO yields despite the high Pd dispersion. In the case of Pd/SiO_2_, the peak for Pd^0^ phase became weaker after the reaction, and the low DHPO yield was obtained. Pd/C(Wako) also showed the lower DHPO yield, and it could be due to the large size of the Pd particles. From these results, the catalyst with low valent and stable Pd^0^ species during the reaction under O_2_ was effective for the DHPO formation.

### 3.7 Reaction Mechanism

**FIGURE 9:**
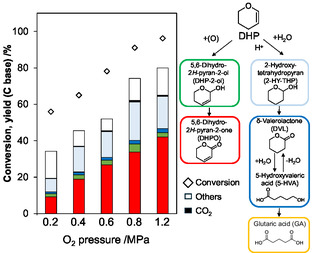
Effect of O_2_ pressure on the DHP oxygenation over Pd/C. Reaction conditions: Pd/C(BP2000) (Pd = 5 wt%) 0.10 g (47 µmol as Pd), NaHCO_3_ 50 µmol, DHP 4.0 mmol, water 10 g, 313 K, 16 h.

The effect of O_2_ pressure on the DHP oxidation with Pd/C catalyst was investigated (Figure 9). The reaction was carried out for 16 h at which the DHPO yield significantly changed in the time course study (*vide supra*). Both the conversion and DHPO yield increased steadily with the increase of O_2_ pressure in the range of 0.2–1.2 MPa, which means that O species is involved in the rate‐determining step.

**FIGURE 10:**
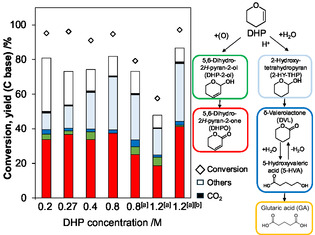
Effect of DHP concentration on the DHP oxygenation over Pd/C. Reaction conditions: Pd/C(BP2000) (Pd = 5 wt%) 47 µmol as Pd, DHP 4.0 mmol, 5 mM NaHCO_3_ aq. 20, 15, 10 or 5 g, ^a^NaHCO_3_ 50 µmol in water 5 g or 3.1 g, O_2_ 0.8 MPa, 313 K, 16 h, ^b^24 h.

The dependence of DHP concentration was next investigated (Figure 10). The concentration was changed by controlling the amount of solvent (5–20 g), while the catalyst amount, DHP amount and NaHCO_3_ concentration were kept constant. The production amount of DHPO was almost unchanged by the DHP concentration, which means very low reaction order with respect to the DHP concentration, and the strong adsorption of DHP on the catalyst surfaces was suggested. The formation amount of the hydration product of DHP (i.e., 2‐HY‐THP) increased with the increase of DHP concentration. This behavior is possibly due to the smaller amount of NaHCO_3_ than that in the standard run in the reaction system under the highly concentrated (smaller solvent amount) conditions. When the reactions with higher DHP concentration were carried out with constant NaHCO_3_/DHP ratio, the DHP conversion gradually decreased, probably because of the higher concentration of NaHCO_3_. At high conversion level in the conditions of higher DHP and NaHCO_3_ concentrations, slightly lower yield (42%) than that in the standard conditions (52%) was obtained.

The effect of solvent was further investigated. While green and safe water solvent was the standard one in our system, we further tested acetonitrile and *tert*‐butyl alcohol solvents and solventless conditions with Pd/C(BP2000) catalyst (Supporting Information Table S8). The hydration of DHP was suppressed in these nonaqueous conditions; however, the conversion of DHP was much lower than that in water solvent. DHPO was not produced under solventless conditions or in acetonitrile solvent. On the other hand, DHPO was detected in *tert*‐butyl alcohol solvent, and at long reaction time under mild conditions the DHPO yield reached 42%. The addition reaction of *tert*‐butyl alcohol to DHP also occurred as a side reaction, which limited the DHPO yield. The results indicate that water solvent is the most effective but is not directly involved in the formation of DHPO.

**FIGURE 11:**
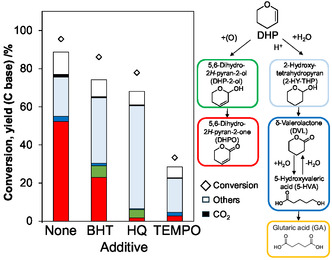
Effect of addition of various radical scavengers on the DHP oxygenation with Pd/C catalyst. Reaction conditions: Pd/C(BP2000) (Pd = 5 wt%) 47 µmol as Pd, NaHCO_3_ 50 µmol, additive (BHT, HQ, or TEMPO) 1.0 mmol, DHP 4.0 mmol, water 10 g, O_2_ 0.8 MPa, 313 K, 24 h.

The additions of radical scavengers were tested for the DHP oxidation with the Pd/C catalyst (Figure 11). The tested radical scavengers were 2,6‐di‐*tert*‐butyl‐*p*‐cresol (BHT), hydroquinone (HQ), and 2,2,6,6‐tetramethylpiperidine 1‐oxyl (TEMPO). All radical scavengers lowered the DHPO yield, suggesting that the production step of DHPO from DHP involves a radical intermediate. However, the decrease of DHPO formation amount was not large in the case of BHT, and the mechanism does not seem to be a long radical chain reaction like free radical autoxidation. In the case of HQ, the hydration of DHP more proceeded because of the higher acidity of HQ, which may also contribute to the suppression of DHPO formation. The detection of the adduct of radical scavengers was unsuccessful, probably because of the low solubility in water solvent and adsorption on carbon support.

**FIGURE 12:**
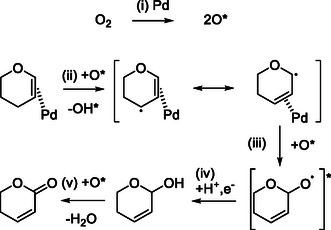
Proposed reaction mechanism.

For Pd‐catalyzed oxidation with O_2_, typical proposed mechanisms involve the coordination of substrate to Pd^2+^ species as the key step [38, 39], including the well‐known mechanism of the Wacker process [40]. The reaction starts with dispersed Pd^2+^ species, and the cycle does not involve a radical intermediate. However, in our system, the metallic Pd phase seems to be important, and the suppression by a radical scavenger was observed. In addition, water molecule is not directly involved in this system unlike the Wacker process where water addition is one of key elementary reactions. Based on the reaction results, we proposed the reaction mechanism of DHP to DHPO as shown in Figure 12. First, O_2_ is adsorbed on Pd, and reactive O species are formed (step (i) in Figure 12). Next, the reactive O species abstract the hydrogen at the C4 position of DHP adsorbed on Pd, and an allyl radical intermediate is produced (step (ii)), which is the rate‐determining step. The adsorption of alkenes on the metallic Pd surface is well known in the chemistry of metal‐catalyzed hydrogenation [41]. Involvement of oxygen atom on metallic Pd has been proposed for oxidation of highly reactive alcohols [42, 43, 44]. After the addition of oxygen (step (iii)), the intermediate receives an electron and proton from Pd metal and water solvent, respectively, and DHP‐2‐ol is formed (step (iv)). Finally, DHPO is formed by oxidative dehydrogenation of the ‐OH group of DHP‐2‐ol (step (v)). The step (v) is quite fast because the C—H bond is highly activated by the two oxygen atoms and C=C group bonded to the carbon atom.

## 4 Conclusions

We reported the oxygenation of DHP to DHPO in high yields (up to 52%) over the Pd/C(BP2000) catalyst using O_2_ as an oxidant in a H_2_O solvent. The addition of base like NaHCO_3_ is effective to suppress undesired hydration of DHP and increases DHPO yield. The Pd/C(BP2000) catalyst is reusable and can be regenerated by the treatment with H_2_. The characterization of Pd catalysts posits that the catalysts with low valent and stable Pd^0^ phase during the reaction, such as Pd/C and Pd/BN, are effective for the DHPO formation. We propose the reaction mechanism involving the removal of allylic hydrogen of DHP adsorbed on Pd species with activated oxygen species. This work offers a new green production route of a biomass‐derived chemical.

## Supporting Information

Additional supporting information can be found online in the Supporting Information section. **Supporting Fig. S1**: GC chart of the typical reaction mixture. **Supporting Fig. S2**: (A) Total ion chromatogram recorded by GC‐MS (EI) for the reaction mixture of oxidation of DHP, and mass spectra of the peaks at (B) 5.9 min, (C) 6.1 min, (D) 12.3 min, (E) 12.6 min, (F) 12.9 min, (G) 15.0 min, (H) 18.8 min. **Supporting Fig. S3**: XRD patterns of ZnFe_2_O_4_@g‐Al_2_O_3_ (ZF@g‐Al_2_O_3_). (A) Sample prepared by us. (B) Results in reference [S6] (reprinted from reference [S6]; Copyright 2023 by the authors of reference [S6]; under the terms and conditions of the Creative Commons Attribution (CC BY) license (https://creativecommons.org/licenses/by/4.0/)). **Supporting** **Table S1**: Previous studies on oxidation of 3,4‐dihydro‐2*H*‐pyran (DHP). **Supporting** **Table S2**: List of reagents used in this study. **Supporting Table S3**: List of supports tested in this study. **Supporting Table S4**: Effect of addition of NaHCO_3_ on the Oxidation of DHP with Pd/C catalyst (Details of Figure 2). **Supporting Table S5**: Time course of DHP oxidation with Pd/C catalyst (Details of Figure 3). **Supporting Table S6**: Reproduction experiment of the report by Abuduh et al. [S6]. **Supporting Table S7**: Dispersion and performance of Pd catalysts. **Supporting Table S8**: Solvent effect on Pd/C(BP2000)‐catalyzed DHP oxidation.

## Funding

Japan Society for the Promotion of Science (KAKENHI 23K20034, KAKENHI 23H05404, KAKENHI 23K26451).

## Conflicts of Interest

The authors declare no conflicts of interest.

## Supporting information

Supplementary Material

## Data Availability

The data that support the findings of this study are available from the corresponding author upon reasonable request.
