# Supplementary material for: Selective Oxygenation of 3,4‐Dihydro‐2H‐Pyran to 5,6‐Dihydro‐2H‐Pyran‐2‐One With Pd/C Catalyst and Molecular Oxygen
Source: ChemSusChem. 2026 Mar 29;19(7):e202502360. doi: 10.1002/cssc.202502360 (PMC13033343; doi:10.1002/cssc.202502360)
Supplement: Supplementary file 1 — Supplementary Material [file CSSC-19-e202502360-s001.pdf]

Supporting Information

Selective oxygenation of 3,4-dihydro-2*H*-pyran to 5,6-dihydro-2*H*-pyran-2-one with Pd/C catalyst and molecular oxygen

Naoki Kawabata,[a] Mizuho Yabushita,[a][b] Keiichi Tomishige,*[a][b][c] and Yoshinao Nakagawa*[a][b]

[a] Department of Applied Chemistry, Graduate School of Engineering, Tohoku University

6-6-07 Aoba, Aramaki, Aoba-ku, Sendai, Miyagi 980-8579, Japan

[b] Research Center for Rare Metal and Green Innovation, Tohoku University,

468-1 Aoba, Aramaki, Aoba-ku, Sendai, Miyagi, 980-0845, Japan

[c] Advanced Institute for Materials Research (WPI-AIMR), Tohoku University,

2-1-1 Katahira, Aoba-ku, Sendai, Miyagi, 980-8577, Japan

[*] Email: tomishige@tohoku.ac.jp

yoshinao@erec.che.tohoku.ac.jp

Table S1. Previous studies on oxidation of 3,4-dihydro-2*H*-pyran (DHP).

Entry	Product	Year of publication	Catalyst	Oxidant	Solvent	Temp. [K]	Conv. [%]	Yield [%]	Ref.
1	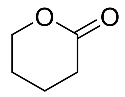	2025	Cu/SiO_2	-	Tetrahydropyran	423	90[a]	76	S1
2		2022	Ruthenium acridine-based PNP(Ph)-type complex	-	H_2O + Dioxane (1 : 2)	423	-	72	S2
3		2011	Titanosilicate-1	H_2O_2 (1 equiv.)	Water	343	39	20	S3
4		1977	-	PCC (2 equiv.)	CH_2Cl_2	r.t.	-	90	S4
5	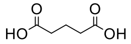	1957	-	HNO_3 (4.7 equiv.)	H_2O	273−303	-	75	S5
6	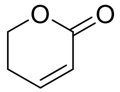	2023	$ZnFe_2O_4$@γ-Al_2O_3	O_2	None	358	49	35[b]	S6
7		2017	$Rh_2(cap)_4$	TBHP (5 equiv.)	CH_2Cl_2	r.t.	100	37	S7
8		1989	-	PDC and TBHP (2 and 3 equiv.)	CH_2Cl_2	273	-	56	S8
9		1973	Rose bengal (+*hv*)	O_2	CH_2Cl_2	283	-	27	S9
10		2025	Pd/C(Pd=5 wt%, C=BP2000)	O_2	H_2O	313	96	52	This work

PCC: Pyridinium chlorochromate, PDC: Pyridinium dichromate, TBHP: *tert*-Butyl hydroperoxide. [a] 2-Hydroxy-tetrahydropyran was used as substrate.

[b] Reproduction experiment was unsuccessful: see Table S6.

Table S2. List of reagents used in this study.

Reagent	Supplier	Purity [%]
$Pd(NO_3)_2$	FUJIFILM Wako Pure Chemical Co.	99.9
3,4-Dihydro-2*H*-pyran	Tokyo Chemical Industry Co., Ltd.	> 97.0
1,2-Diethoxyethane	FUJIFILM Wako Pure Chemical Co.	> 98.0
Tetrahydrofuran	FUJIFILM Wako Pure Chemical Co.	> 99.5
5,6-Dihydro-2*H*-pyran-2-one	Tokyo Chemical Industry Co., Ltd.	> 95.0
δ-Valerolactone	Tokyo Chemical Industry Co., Ltd.	> 98.0
2,6-Di-*tert*-butyl-*p*-cresol	Tokyo Chemical Industry Co., Ltd.	> 99.0
Hydroquinone	Tokyo Chemical Industry Co., Ltd.	> 99.0
2,2,6,6-Tetramethylpiperidine 1-oxyl	Tokyo Chemical Industry Co., Ltd.	> 98.0
tert-butyl alcohol	FUJIFILM Wako Pure Chemical Co.	> 99.0
Acetonitrile	Kanto Chemical Co., Inc.	> 99.8
Fe_3O_4	FUJIFILM Wako Pure Chemical Co.	> 95.0
MnO_2	FUJIFILM Wako Pure Chemical Co.	> 99.5
CuO	FUJIFILM Wako Pure Chemical Co.	> 95.0
V_2O_5	Tokyo Chemical Industry Co., Ltd.	> 98.0
$H_3PMo_{12}O_{40} \cdot nH_2O$	Tokyo Chemical Industry Co., Ltd.	-
$H_5PV_2Mo_{10}O_{40} \cdot 25H_2O$	Nippon Inorganic Colour & Chemical Co., Ltd.	-
Pt/C(Pt=10 wt%)	Sigma-Aldrich Co.	BET surface area 437 m^2 g^{-1}
Pd/C(Pd=5 wt%)	FUJIFILM Wako Pure Chemical Co.	BET surface area 501 m^2 g^{-1}

Table S3. List of supports tested in this study.

Support	Supplier	BET surface area [$m^2\ g^{-1}$]	Detail information
Al_2O_3	Catalysis Society of Japan	176	JRC-ALO-2, calcined at 973 K for 3 h.
BN	MARUKA Co.	7.5	-
BP2000	Cabot Co.	1280	Black Pearls 2000
CeO_2	Daiichi Kigenso Kagaku Kogyo Co., Ltd.	78	HS, calcined at 873 K for 3 h.
SiO_2	FUJI SILYSIA Chemical Ltd.	402	CARIACT
TiO_2 (P25)	Aeroxide Co.	50	P25
XC72R	Cabot Co.	208	VULCAN XC72R
ZrO_2	Daiichi Kigenso Kagaku Kogyo Co., Ltd.	39	RC-100

Table S4 Effect of addition of $NaHCO_3$ on the Oxidation of DHP with Pd/C catalyst (Details of Fig. 2)

Entry	Catalyst	Amount of base [μmol]	pH before(after)	Conv. [%]	Yield [%]								C. B.[%]
					DHPO	DHP-2-ol	2-HY-THP	DVL+ 5-HVA	GA	CO	CO_2	Others	
1	None	0	7.3 (3.9)	82	<1	<1	69	<1	<1	<1	<1	4	91
2		50	8.4	31	<1	<1	6	<1	<1	<1	<1	8	83
3	Pd/C(BP2000)	0	7.0 (3.0)	92	22	<1	30	22	<1	<1	<1	6	89
4		25	8.3	95	45	<1	24	7	<1	<1	2	9	92
5		50	8.4 (3.9)	96	52	<1	21	3	<1	<1	1	11	93
6		100	8.4	97	52	2	19	4	<1	<1	<1	10	91
7		200	8.4	88	28	2	13	5	<1	<1	<1	26	86
8		1000	8.3 (8.4)	48	4	<1	13	3	<1	<1	<1	15	86
9		50[a]	11	72	17	<1	11	2	<1	<1	<1	21	79

Reaction conditions: Pd/C(BP2000)(Pd=5 wt%) 0.10 g (47 μmol as Pd), $NaHCO_3$ 0-1000 μmol or [a]Na_2CO_3 50 μmol, DHP 4.0 mmol, water 10 g, O_2 0.8 MPa, 313 K, 24 h.

DHPO: 5,6-dihydro-2*H*-pyran-2-one; DHP-2-ol: 5,6-dihydro-2*H*-pyran-2-ol; 2-HY-THP: 2-hydroxytetrahydropyran; DVL: δ-valerolactone; 5-HVA: 5-hydroxyvaleric acid; GA: glutaric acid.

Table S5 Time course of DHP oxidation with Pd/C catalyst (Details of Fig. 3)

Entry	Reaction time [h]	Conv. [%]	Yield [%]								C. B.[%]
			DHPO	DHP-2-ol	2-HY-THP	DVL+ 5-HVA	GA	CO	CO_2	Others	
1	0	25	<1	<1	<1	<1	<1	<1	<1	8	84
2	2	36	2	1	1	<1	<1	<1	<1	9	78
3	4	35	3	2	2	2	<1	<1	<1	16	90
4	8	60	11	3	9	1	<1	<1	<1	19	84
5	16	91	34	5	21	2	<1	<1	<1	12	83
6	20	97	48	3	20	3	<1	<1	<1	17	95
7	24	96	52	<1	21	3	<1	<1	1	11	93
8	48	97	51	<1	17	11	<1	<1	1	15	98

Reaction conditions: Pd/C(BP2000)(Pd=5 wt%) 0.1 g (47 μmol as Pd), $NaHCO_3$ 50 μmol, 3,4-dihydro-2*H*-pyran 4.0 mmol, water 10 g, O_2 0.8 MPa, 313 K.

Table S6 Reproduction experiment of the report by Abuduh *et al.* [S6]

Entry	Catalyst	O_2 pressure [MPa]	Time [h]	Conv. [%]	Yield [%]								C. B. [%]
					DHPO	DHP-2-ol	2-HY-THP	DVL+ 5-HVA	GA	CO	CO_2	Others	
1	None	0.15	6	58	2	<1	<1	1	<1	<1	<1	46	92
2	None	0.35	6	71	3	1	<1	1	<1	<1	<1	48	84
3	$ZnFe_2O_4@\gamma\text{-}Al_2O_3$ [a]	0.15	6	59	3	<1	4	<1	<1	<1	<1	29	79
4	$ZnFe_2O_4@\gamma\text{-}Al_2O_3$ [a]	0.35	6	77	3	<1	4	1	<1	<1	<1	36	67
5	$ZnFe_2O_4@\gamma\text{-}Al_2O_3$ [b]	0.15	6	49	35								
6	$ZnFe_2O_4@\gamma\text{-}Al_2O_3$ [b]	0.35	12	38	23								

Reaction conditions: DHP 10 mL, $ZnFe_2O_4@\gamma\text{-}Al_2O_3$ 0 or 0.5 g, O_2 0.15 or 0.35 MPa[c], 358 K, 6 or 12 h.

[a] Prepared by ours. [b] Results in ref. [S6]. [c] O_2 was repressurized every 30 minutes.

Table S7 Dispersion and performance of Pd catalysts

Entry	Catalyst	D^a[%]	Conv. [%]	Yield [%]								C. B.[%]
				DHPO	DHP-2-ol	2-HY-THP	DVL+ 5-HVA	GA	CO	CO_2	Others	
1	Pd/CeO_2	32	32	2	1	3	<1	<1	<1	<1	8	83
2	Pd/Al_2O_3	19	44	3	1	6	1	<1	<1	<1	10	78
3	Pd/ZrO_2	16	80	9	8	10	2	<1	<1	<1	25	74
4	Pd/TiO_2	7.0	80	13	10	8	2	<1	<1	<1	22	74
5	Pd/SiO_2	13	68	7	7	12	2	<1	<1	<1	19	80
6	Pd/BN	7.9	94	48	2	17	3	<1	<1	<1	15	93
7	Pd/C(Wako)	10	86	13	7	14	4	<1	<1	<1	31	83
8	Pd/C(XC72R)	3.0	78	15	9	11	2	<1	<1	<1	22	83
9	Pd/C(BP2000)	0.8	91	34	3	20	2	<1	<1	<1	19	88

Reaction conditions: Pd/Support(Pd=5 wt%) 0.10 g (47 μmol as Pd), $NaHCO_3$ 50 μmol, DHP 4.0 mmol, water 10 g, O_2 0.8 MPa, 313 K, 16 h.

[a] Dispersion (%)=[amount (mol) of adsorbed CO /total amount (mol) of Pd]$\times$100.

Table S8 Solvent effect on Pd/C(BP2000)-catalyzed DHP oxidation

Entry	Solvent	Temp. [K]	Time [h]	Conv. [%]	Yield [%]										C. B. [%]
					DHPO	DHP-2-ol	2-HY-THP	DVL+ 5-HVA	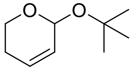 [d]	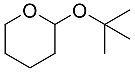 [e]	GA	CO	CO_2	Others	
1[a]	None	333	4	21	<1	<1	3	<1	<1	<1	<1	<1	<1	2	85
2[b]	H_2O	333	24	100	28	<1	6	45	<1	<1	2	<1	<1	22	103
3[b]	CH_3CN	333	24	2	<1	<1	1	<1	<1	<1	<1	<1	<1	2	101
4[b]	^{t}BuOH	333	24	31	4	1	7	<1	5	15	<1	<1	<1	1	102
5[c]	^{t}BuOH	313	48	94	42	<1	18	<1	3	31	<1	<1	<1	6	106

[a] Reaction conditions: Pd/C(BP2000) (Pd=5 wt%) 0.10 g (47 μmol as Pd), DHP 30 mmol, O_2 0.8 MPa, 333 K, 4 h.

[b] Reaction conditions: Pd/C(BP2000) (Pd=5 wt%) 0.10 g (47 μmol as Pd), DHP 4 mmol, solvent 5 g, O_2 0.8 MPa, 333 K, 24 h.

[c] Reaction conditions: Pd/C(BP2000) (Pd=5 wt%) 0.10 g (47 μmol as Pd), DHP 2 mmol, solvent 5 g, O_2 0.8 MPa, 313 K, 48 h.

[d] MS of 2-(*tert*-butoxy)-5,6-dihydro-2*H*-pyran: *m*/*z* 156 (trace, $[M]^+$), 141 (trace, $[M-CH_3]^+$), 126 (trace, $[M-CH_2O]^+$), 100 (20%), 83 (100%, $[C_5H_7O]^+$), 70 (6%), 57 (17%), 55 (13%), 41 (10%).

[e] MS of 2-(*tert*-butoxy)-tetrahydropyran: *m*/*z* 158 (trace, $[M]^+$), 143 (trace, $[M-CH_3]^+$), 103 (25%), 101 (8%), 85 (51%, $[C_5H_9O]^+$), 67 (10%), 57 (100%, $[C_4H_9]^+$), 56 (32%), 43 (10%), 41 (31%).

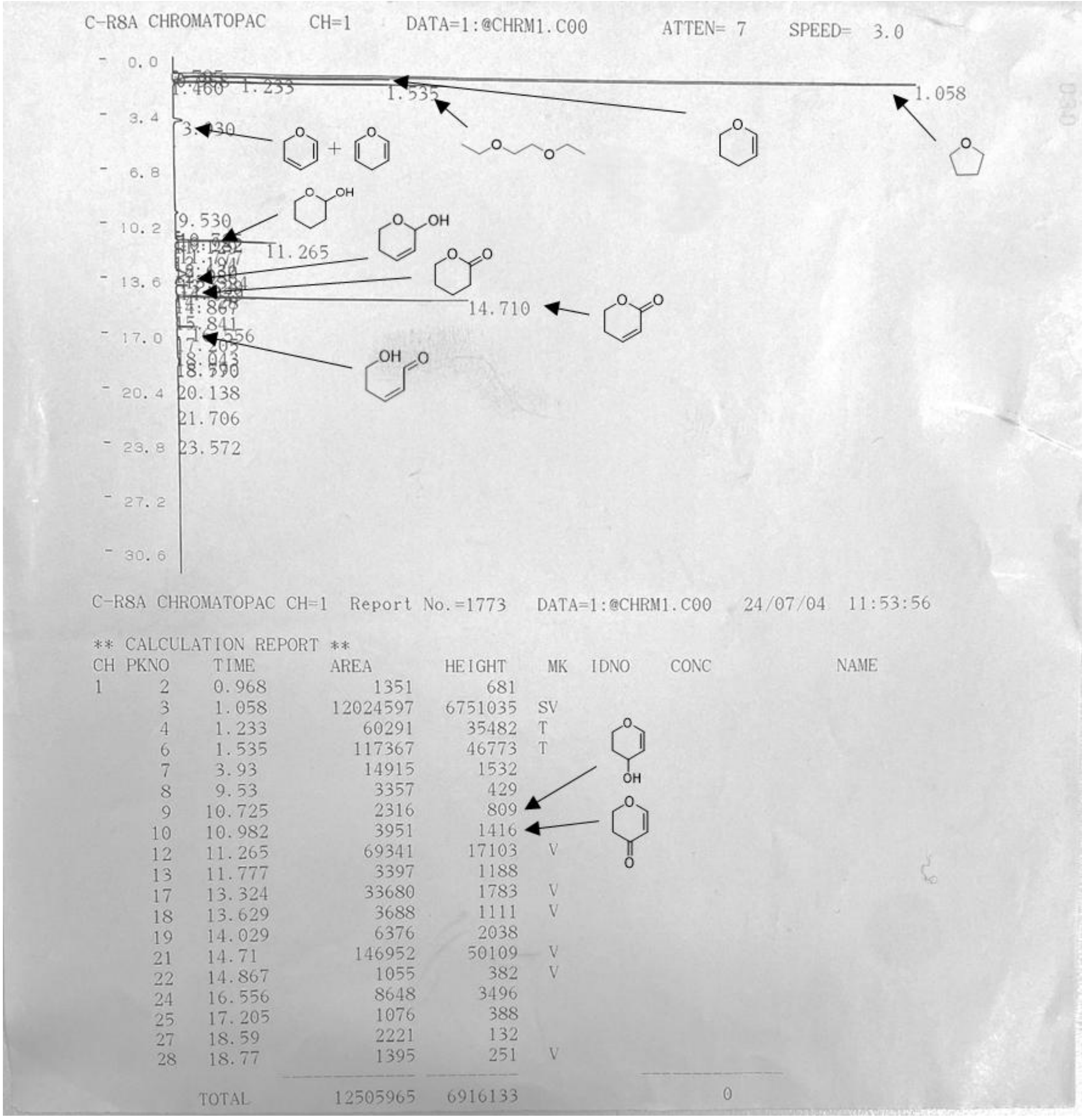

C-R8A CHROMATOPAC CH=1 Report No.=1773 DATA=1:@CHRM1.C00 24/07/04 11:53:56

** CALCULATION REPORT **

CH	PKNO	TIME	AREA	HEIGHT	MK	IDNO	CONC	NAME
1	2	0.968	1351	681				
	3	1.058	12024597	6751035	SV			
	4	1.233	60291	35482	T			
	6	1.535	117367	46773	T			
	7	3.93	14915	1532				
	8	9.53	3357	429				
	9	10.725	2316	809				
	10	10.982	3951	1416				
	12	11.265	69341	17103	V			
	13	11.777	3397	1188				
	17	13.324	33680	1783	V			
	18	13.629	3688	1111	V			
	19	14.029	6376	2038				
	21	14.71	146952	50109	V			
	22	14.867	1055	382	V			
	24	16.556	8648	3496				
	25	17.205	1076	388				
	27	18.59	2221	132				
	28	18.77	1395	251	V			
	TOTAL		12505965	6916133			0	

Fig. S1 GC chart of the typical reaction mixture

Reaction conditions: Pd/BP2000(Pd=5 wt%) 0.1 g (47 μmol as Pd), $NaHCO_3$ 50 μmol, DHP 4.0 mmol, water 10 g, O_2 0.8 MPa, 313 K, 16 h.

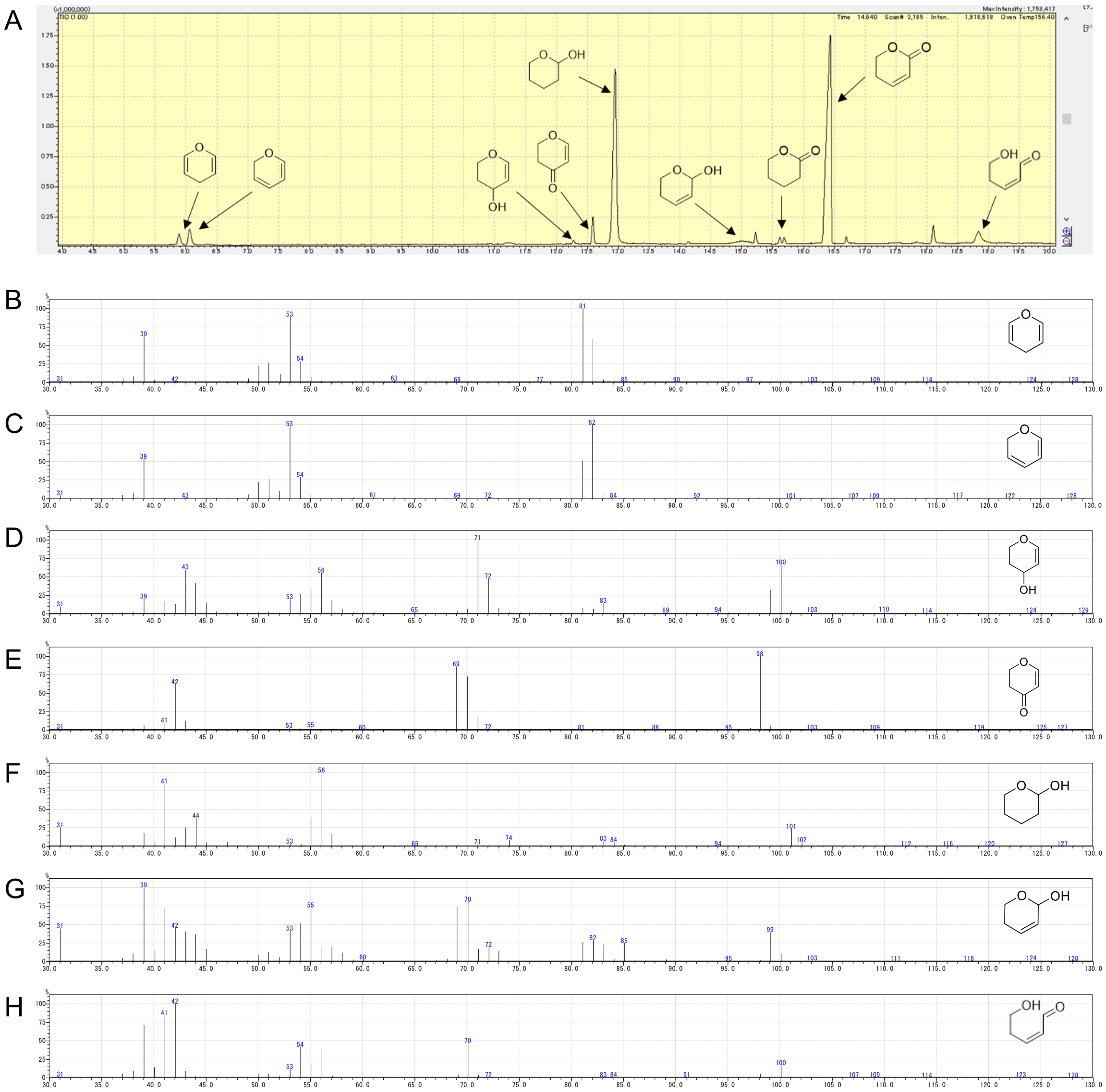

Fig. S2 (A) Total ion chromatogram recorded by GC-MS (EI) for the reaction mixture of oxidation of DHP, and mass spectra of the peaks at (B) 5.9 min, (C) 6.1 min, (D) 12.3 min, (E) 12.6 min, (F)12.9 min, (G)15.0 min, (H)18.8 min.

Reaction conditions: Pd/BP2000(Pd=5 wt%) 0.1 g (47 μmol as Pd), $NaHCO_3$ 50 μmol, DHP 4.0 mmol, water 10 g, O_2 0.8 MPa, 313 K, 16 h.

Column for GC-MS: HP-FFAP column (ø0.25 mm × 30 m).

Assignment for mass spectra:

[Fig. S2B] 4H-pyran (M=82 g mol^{-1}), [Fig. S2C] 2H-pyran (M=82 g mol^{-1}), [Fig. S2D] 3,4-dihydro-2*H*-pyran-4-ol (M=100 g mol^{-1}),[Fig. S2E] 3,4-dihydro-2*H*-pyran-4-one, [Fig. S2F] 2-hydroxy-tetrahydro-pyran (M=102 g mol^{-1}), [Fig. S2G] 5,6-dihydro-2*H*-pyran-2-ol (M=100 g mol^{-1}), [Fig. S2H] 5-hydroxy-2-pentenal (M=100 g mol^{-1}).

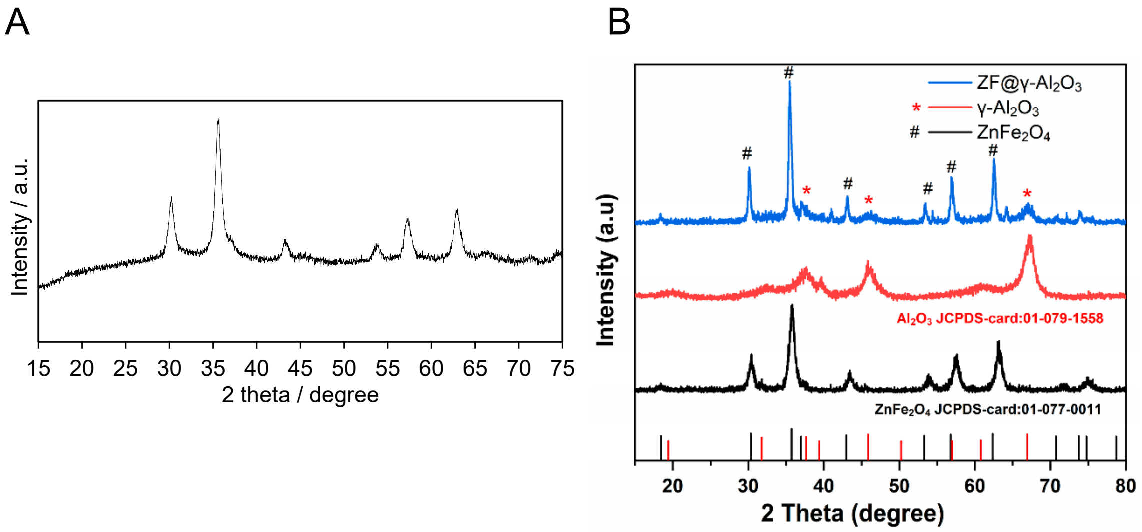

Fig. S3 XRD patterns of $ZnFe_2O_4$@γ-Al_2O_3 (ZF@γ-Al_2O_3). (A) Sample prepared by us. (B) Results in ref. [S6] (reprinted from ref. [S6]; Copyright 2023 by the authors of ref. [S6]; under the terms and conditions of the Creative Commons Attribution (CC BY) license (https://creativecommons.org/licenses/by/4.0/)).

Preparation procedure for our $ZnFe_2O_4$@γ-Al_2O_3:

4.04 g ferric nitrate nonahydrate, 1.1 zinc acetate dihydrate and 1.1 g γ-alumina (JRC-ALO-2) (1:2 molar ratio of Zn:Fe) were added to a 250 mL beaker containing 30 mL distilled water. The mixture was stirred at 313 K for 1 h, and then NaOH aq. was added drop-wisely until pH 8.5. The resulting precipitate was stirred at 348 K for further 5 h. After 12 h of aging, the precipitate was filtered, washed, and dried overnight at 348 K. Calcination at 873 K was then performed on the catalyst precursor for 5 h. The formation of $ZnFe_2O_4$ was confirmed by XRD measurement (Fig. S3A), and the crystallite size was even smaller than that in the original report (Fig. S3B).

References

[S1] R. G. Dastidar, J. E. Chavarrio, Z. Jiang, D. J. McClelland, M. Mavrikakis, G. W. Huber, *Appl. Catal. B*, **2025**, 360, 124519.

[S2] S. Kar, J. Luo, M. Rauch, Y. Diskin-Posner, Y. Ben-David, D. Milstein, *Green Chem.*, **2022**, 24, 1481–1487.

[S3] M. Sasidharan, A. Bhaumik, *J. Mol. Catal. A*, **2011**, S138111691100046X.

[S4] G. Piancatelli, A. Scettri, M. D'Auria, *Tetrahedron Lett.*, **1977**, 18, 3483–3484.

[S5] J. English, J. E. Dayan, *Org. Synth.*, **1957**, 37, 47.

[S6] N. A. Y. Abduh, A. A. Al-Kahtani, M. S. Amer, T. S. Algarni, A.-B. Al-Odayni, *Molecules*, **2023**, 28, 7192.

[S7] Y. Yu, R. Humeidi, J. R. Alleyn, M. P. Doyle, *J. Org. Chem.*, **2017**, 82, 8506–8513.

[S8] N. Chidambaram, K. Satyanarayana, S. Chandrasekaran, *Tetrahedron Lett.*, **1989**, 30, 2429–2432.

[S9] E. C. Blossey, D. C. Neckers, A. L. Thayer, A. P. Schaap, *J. Am. Chem. Soc.*, **1973**, 95, 5820–5822.
